# Current Directions of Selected Plant-Origin Wastes’ Valorization in Biotechnology of Food Additives and Other Important Chemicals

**DOI:** 10.3390/foods14060954

**Published:** 2025-03-11

**Authors:** Dominika Popielarz, Pavol Farkaš, Anna Bzducha-Wróbel

**Affiliations:** 1 Department of Food Biotechnology and Microbiology, Institute of Food Sciences, Warsaw University of Life Sciences, Nowoursynowska 159C Street, 02-787 Warsaw, Poland; dominika_popielarz@sggw.edu.pl; 2 Department of Glycobiotechnology, Institute of Chemistry Slovak Academy of Sciences, Dúbravská cesta 9, SK-845 38 Bratislava, Slovakia; pavol.farkas@savba.sk

**Keywords:** plant-origin wastes, food processing waste, ecology, valorization, microorganisms, recycling

## Abstract

Environmental pollution and the accumulation of industrial waste are increasingly serious issues that impose financial burdens on businesses and pose threats to ecosystems. As industrial production continues to grow, the volume of waste generated by humanity is rising, leading to a heightened need to search for effective waste management and recycling methods. One promising approach is the concept of a circular economy, where industrial waste, including agricultural and food processing waste, is transformed into new products. The goal is to maximize the utilization of natural resources, particularly in food production. This article presents various concepts for utilizing specific types of plant-based waste, particularly lignocellulosic, pectin, and starch wastes, in biotechnological processes aimed at producing value-added food ingredients with a technological function. The literature clearly shows that this waste can be effectively used in the cultivation of different microorganisms to produce enzymes, polyols, oligosaccharides, carboxylic acids, and biopolymers, among other products. However, further research is needed to explore more efficient and environmentally friendly methods, especially in the utilization of lignocellulose in biotechnology. This research shows knowledge gaps in existing discussed solutions.

## 1. Introduction

The accumulation of industrial waste and the resulting environmental burden stemming from its accumulation are of increasing economic and ecological concern. The costs of disposing of post-production residues continue to escalate, constituting a substantial financial burden for business. Additionally, environmentalists are sounding the alarm about the degree of environmental pollution. The environment can no longer absorb the increasing volume of generated waste. Therefore, it is imperative to seek opportunities for its reutilization and explore new recycling solutions that can alleviate environmental strain and potentially yield additional profits for business [1,2].

The agri-food industry, as one of the largest sectors of industry, has a direct impact on the environment, generating significant amounts of waste across many stages, from primary production to raw material processing, as well as the substantial volumes of packaging waste and food wastage. The global population growth has led to increased demand for food [3], translating into larger quantities of generated waste. The direction of agricultural and food industry development holds particular significance in addressing environmental issues due to the complexity and scale of production. Sustainable development strategies are not a novel concept, as they have been in practice for years. Despite the initiation of desirable changes in agriculture, they remain insufficient in meeting environmental needs. In December 2019, the European Commission issued a communication on the European Green Deal strategy [4], aimed at initiating further international actions to achieve specified climate and environmental goals [2]. Both national and international legislation indicate that waste prevention and minimization, along with valorization of byproducts, are key strategies for an effective waste management system. Satisfying the growing human demand for nutrients can be achieved through the creation of a circular economy, in which food components and waste from agricultural production and food processing are transformed into new products [3]. In recent years, lignocellulosic waste has garnered significant interest due to global climate change and diminishing fossil fuel reserves [5]. It is abundantly generated within the forestry economy. However, apart from conventional sources such as woody and herbaceous plants, lignocellulose is also a byproduct of agri-food processing [6]. The primary challenge in managing these wastes lies in their proper storage and disposal [7,8,9,10]. Currently, lignocellulosic biomass from these sources is mostly used as fertilizer, incinerated for energy production, or treated as waste [11]. Waste pectin generated in the food industry is typically discarded due to its seasonality and susceptibility to spoilage; this poses a problem for both the processing industry and pollution monitoring agencies. Consequently, these residues can create environmental issues, especially concerning water pollution. This is attributed to the presence of biomaterials, such as fruit peels rich in sugars, which can stimulate aerobic bacteria to decompose biodegradable organic substances, transforming them into products such as carbon dioxide, nitrates, sulfates, and phosphates in water. Therefore, it is essential to implement appropriate methods enabling the utilization of these wastes for conversion into value-added products [12,13].

Starchy vegetable wastes also constitute a significant aspect of contemporary waste management challenges. They are frequently generated in the agricultural and food processing sectors, arising as byproducts from both plant crops and processed items. In recent years, with the increasing emphasis on proper and healthy nutrition, there has been a discernible shift in the consumption patterns of fruits and vegetables. These changes have, in turn, led to a heightened demand for these products, consequently contributing to an increase in waste. The Freshfel Europe Consumption Monitor report, based on official EUROSTAT and FAOSTAT statistics, reveals that the average consumption of fruits and vegetables in the EU increased to 364.58 g/day/capita in 2021. This signifies a growth of 2.19% compared to 2020 and 1.27% above the average from the previous five years. Additionally, the size of the fresh fruits and vegetables market in the EU-27 also experienced growth, reaching 74.354.475 tons in 2021 [14]. Therefore, proper disposal of starch, pectin, and lignocellulose waste is of crucial importance in addressing environmental issues and maximizing the potential value derived from these bioresources.

The article seeks to emphasize the importance of transforming plant-origin wastes, especially lignocellulosic, starch, and pectin wastes, into added-value food additives and other important chemicals within the context of the circular economy concept. The review of literature data from 2015 onwards regarding valorization of selected plant-origin wastes in biotechnology was performed to present current ideas but also indications for the future.

### 1.1. Trends in the Literature Search Queries

Analyzing the results of the number of records in the PubMed database for a particular literature query can provide valuable information about the interests of researchers and trends in biotechnology studies related to the raw materials in question. The results of such analysis are shown in Table 1.

Analyzing the gathered results reveals that the literature query associated with biomass raw materials (lignocellulose, lignocellulosic waste, biotechnological use of lignocellulose, starch, biotechnological use of starch, pectin, pectin waste, biotechnological use of pectin, sugar beet pulp, sugar beet pulp waste, straw, straw waste, biotechnological use of straw, brewer’s spent grain, brewer’s spent grain wastes, potato peels, potato peel wastes) generally attract considerable attention within the scientific community, aligning with trends in sustainable development and the exploration of alternative raw materials. Notably, starch emerges as the most prominent literature query, likely owing to its versatility and broad applications across biotechnology, pharmaceuticals, food, and other sectors. In contrast, the literature query directly related to waste indicates that lignocellulosic waste and starch waste garner significantly more attention than pectin waste. The results indicate that there is lower research interest in pectin wastes in a biotechnology context. This suggests that there is potential for further research and experimentation with the use of these raw materials in the biotechnology industry. Although the discrepancy is less pronounced for the literature query directly associated with biotechnological applications (biotechnological use of lignocellulose, starch, and pectin), biotechnological use of lignocellulose still yields the highest number of reports.

A more detailed analysis of the results shown in Table 1 reveals that other lignocellulosic wastes, such as wheat straw, rice straw, quinoa straw, rapeseed straw, sugarcane straw, sugarcane bagasse, oat bran, rice bran, corn cob, corn stover, and corn stalk, also exhibit varying levels of research interest. Among them, wheat straw and rice straw appear to be more extensively studied, as evidenced by a relatively high number of records, while rapeseed straw, quinoa straw, and pineapple cores receive much less attention. This suggests that research on lignocellulosic biomass is largely concentrated on a few key materials, with other potential resources remaining underexplored. A similar trend is observed for fruit and vegetable processing wastes, where some materials, such as pomegranate peel, lemon peel, and orange peel, have been shown research interest, while others, including papaya peel, pineapple peel, and kiwi peel, generate very few records. This suggests that despite their potential as sources of bioactive compounds, certain horticultural wastes remain largely unexamined in biotechnology. The number of records for biotechnological use of specific waste materials is generally much lower than for general waste-related queries, which further confirms that while biomass waste is a well-recognized research area, its direct applications in biotechnology still require further exploration. Notably, despite a relatively high number of records for sugarcane bagasse and sugarcane straw as waste materials, their biotechnological applications remain underrepresented. This indicates a potential area for further research, particularly in the context of sustainable waste valorization. Upon examining the number of records for individual wastes, a notable scarcity of articles on brewer’s spent hops becomes evident, underscoring a significant research gap in the utilization of these waste materials. Although the literature query “brewer’s spent hops” generates very few results, this is an area worthy of further research, especially given that brewery waste is an important resource that can be used in various biotechnology processes.

In conclusion, while the scientific community exhibits considerable interest in biomass-derived waste materials, the analysis of the literature records highlights significant research gaps. Certain waste materials, such as brewer’s spent hops, quinoa straw, rapeseed straw, and various fruit and vegetable peels, remain underexplored despite their potential applications in biotechnology. Addressing these gaps could contribute to the broader implementation of waste valorization strategies and the sustainable utilization of agricultural and industrial byproducts.

### 1.2. General Characteristics of Lignocellulosic, Pectin, and Starch Wastes

**Figure 1:**
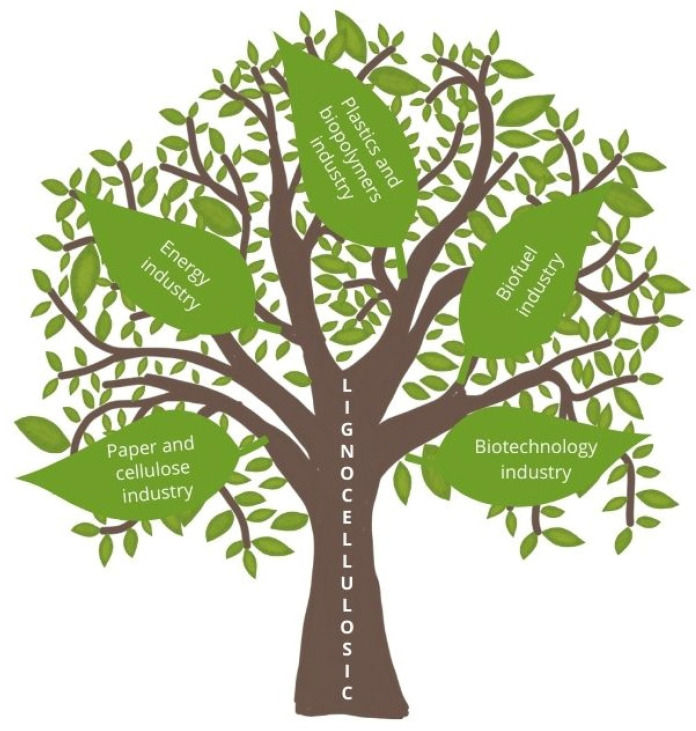
The primary industries where lignocellulose is currently used.

Lignocellulosic biomass is a commonly occurring, renewable biological resource. Due to its availability, diverse composition, and low cost, lignocellulosic biomass is a promising secondary raw material [15]. Lignocellulosic biomass constitutes over 90% of all plant biomass [16]. Currently, lignocellulose is used for biofuel production (bioethanol and biogas) or burned as an environmentally friendly fuel. The biofuel industry relies on lignocellulosic feedstocks to produce second-generation bioethanol and biogas, paving the way for cleaner and more sustainable energy solutions. Meanwhile, the energy industry benefits from lignocellulosic biomass as a renewable and sustainable fuel source, helping to reduce dependence on fossil fuels [17,18]. In addition, lignocellulose is processed into cellulose fibers, nanocellulose, and biocomposites for packaging production and serves as a primary raw material for paper manufacturing [19,20]. In the plastic and biopolymer industry, lignocellulose is gaining attention as a key ingredient in the development of biodegradable plastics and bio-based composites, offering an eco-friendly alternative to traditional petroleum-based materials. In the paper and cellulose industry, lignocellulose plays a fundamental role in producing paper, cardboard, and high-value cellulose derivatives, such as microcrystalline cellulose. Additionally, the biotechnology industry continues to explore innovative ways to harness lignocellulose in microbial fermentation processes, unlocking its potential for producing valuable enzymes, organic acids, and bio-based chemicals used across various sectors. Specific examples of these applications are discussed in Section 2 of this article. The primary industries where lignocellulose is currently used are illustrated in Figure 1; however, they are not sufficient to valorize all available resources. Due to the complex polymeric structure of lignocellulose, in order to use it in biotechnological processes, its hydrolysis is necessary. This process results in the release of simple sugars, such as glucose or xylose, which provide a bioavailable carbon source for microorganisms. Hence, most studies of a biotechnological nature use lignocellulosic waste hydrolysates obtained by pretreatment (physical, chemical, physicochemical, and/or biological) and/or enzymatic hydrolysis. The most commonly used processes for treating lignocellulose are separate hydrolysis and fermentation (SHF), simultaneous saccharification and fermentation (SSF), where saccharification and fermentation processes occur in stages but in a single apparatus, and consolidated bioprocessing (CBP), in which decomposition of lignocellulosic complexes and production of desired chemical compounds occur simultaneously [21]. A side effect of pretreatment is the formation of byproducts (e.g., formic acid, acetic acid, furfural, hydroxymethylfurfural (HMF), and phenolic compounds) derived from lignocellulose, which inhibit microbial and enzymatic biocatalysts [22]. These compounds negatively impact cellular metabolism during the fermentation process. To effectively process lignocellulose, it is necessary to minimize the production of these inhibitors or eliminate them. This can be achieved by optimizing process parameters, using appropriate catalysts or chemical additives, and employing microorganisms or enzymes that are resistant to inhibitors or capable of converting them into less toxic compounds. These measures are designed to increase the efficiency and cost-effectiveness of lignocellulose processing. Typical detoxification methods include, but are not limited to, adsorption with activated carbon, evaporation, or the use of ion exchange resins. Among these, adsorption on activated carbon is commonly used to detoxify acid hydrolysates due to its high efficiency and low cost [23,24,25].

Microorganisms with proper natural phenotypes or those that have been improved through laboratory evolution or microbial metabolic engineering are crucial for the proper productivity and yield of different added-value bioproducts’ production based on lignocellulose hydrolysates. Discussed desired microbial characteristics of biotechnological potential include pentose utilization or co-utilization with hexoses, tolerance to inhibitors and byproducts of lignocellulose pretreatment, thermotolerance, salt resistance, and/or osmo- and xerotolerance [26].

**Figure 2:**
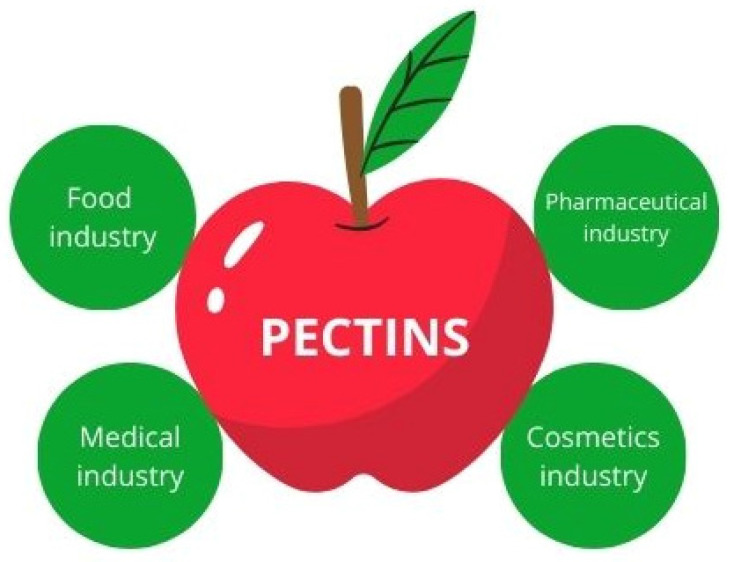
The primary industries where pectins are currently used.

Pectins are heteropolysaccharides commonly found in plants, particularly in cell walls. They are primarily sourced from citrus fruit peels, with lemons, limes, and oranges contributing to 85.5% of the production. A smaller portion, about 14.0%, is extracted from apple pomace, and a negligible amount, 0.5%, is obtained from sugar beet pulp [27]. Pectin, known for its non-toxic nature and desirable properties, finds extensive use in pharmaceuticals and medicine, particularly as a drug delivery system, where its ability to form gels allows for controlled and sustained release of active compounds. It is especially valuable in oral drug formulations, where it helps protect sensitive substances from degradation in the stomach and ensures their release in the intestines. Moreover, pectin-based micro- and nanoparticles are being explored as carriers for targeted drug delivery, including in cancer therapy and gastrointestinal treatments [28]. Recent studies suggest that pectin-based hydrogels and dressings can create a protective barrier over wounds that accelerates healing and reduces the risk of infections. In addition, it exhibits antioxidant properties [29,30]. The food industry is one of the largest consumers of pectin, utilizing its thickening, gelling, stabilizing, and emulsifying properties. It is a key ingredient in jams, jellies, marmalades, and fruit preserves, providing the desired texture and consistency. Furthermore, pectin is used as a fat replacer in low-calorie and plant-based food products, as well as a dietary fiber supplement that supports gut health [31,32,33]. In the cosmetic industry, pectin is commonly incorporated into skincare products such as creams, where it improves product stability and supports emulsion formation. Its natural origin and biocompatibility make it an attractive ingredient for clean-label and eco-friendly cosmetic formulations [34]. Figure 2 illustrates the primary industries that currently actively use pectin; however, these ways of using the indicated type of waste are not sufficient to valorize all available resources. In a sustainable approach to waste management, new methods are being sought to use pectin waste to reduce negative environmental impacts.

**Figure 3:**
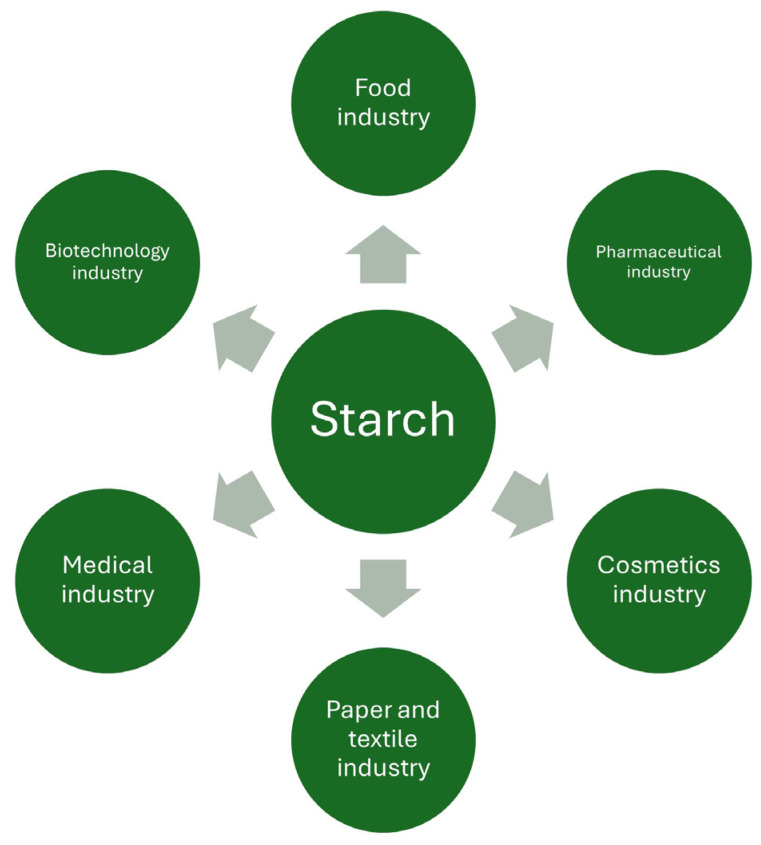
The primary industries where starch is currently used.

**Figure 4:**
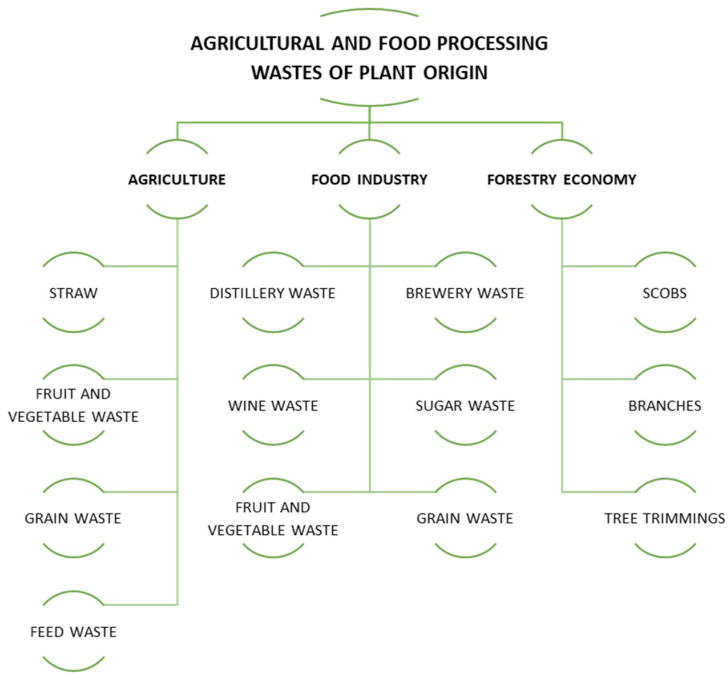
Sources of agro-food waste of plant origin.

Starch is a sugar polymer present in numerous plant roots, crop seeds, and stems. The primary origins of native starch encompass corn (82%), wheat (8%), cassava (5%), and potatoes (5%). Starch plays a crucial role, functioning as the reserve material in all green plants [35]. In the realm of food production, starches are widely used in the manufacturing of baked goods and confectionery as thickening and gelling agents, for example, in the production of jellies [36]. They also enhance the viscosity and structural quality of dairy products, such as yogurts [37]. A recent trend in starch applications is its use in packaging, particularly in the formation of biopolymeric films [38,39]. Beyond the food sector, various non-food industries harness starch for paper production and processing [40], adhesive manufacturing [41], and textiles, where it improves fabric performance [42]. In the cosmetics industry, starch serves as a thickening and stabilizing agent, ensuring a smooth texture in products. Recent studies also highlight its potential as a substitute for talc in powdered formulations [43]. Figure 3 illustrates the primary industries that currently use starches. The properties of starch waste can vary depending on its source and specific industrial or manufacturing processes. The source of energy contained in starchy waste is starch, so regardless of its origin, the production of other compounds must be preceded by hydrolysis (acid or enzymatic) to break down and decompose amylose molecules. It is transformed into dextrins and eventually into glucose. The latter can then be utilized by microorganisms. To achieve high yields of starch hydrolysis, it is extremely important to optimize pretreatment and fermentation conditions [44,45]. This provides very wide possibilities for biotechnological valorization of the above-mentioned type of polymers from starch raw materials unsuitable for consumption. Figure 4 shows example sources of agro-food waste of plant origin.

### 1.3. Characterization of Selected Lignocellulosic, Pectin, and Starch Wastes

In order to better understand the directions for the use of plant-based wastes, the cited articles discuss the compositions of selected wastes, including those that have been most frequently used by researchers in biotechnological processes.

Brewer’s spent grain (BSG) is a major byproduct of the brewing industry. Fresh BSG is currently used as animal feed; however, due to the abundance of nutrients found in its composition (Table 2), it can be used in biosynthesis to produce value-added products [46].

Another significant waste in beer production is brewer’s spent hops, which are obtained by extracting hop oil from hop cones. Residual hops are primarily employed as fertilizer or added to compost. In contrast to yeasts and used grains, spent hops have not found broader use in animal nutrition because of their bitter flavor [47]. The composition of brewer’s spent hops is shown in Table 2.

Sugar production from sugar beets leads to the formation of significant amounts of sugar beet pulp. It is a byproduct whose main components are celluloses, hemicelluloses, and pectin, at a level of 20–36% each, making it a potential source of nutrients (Table 2). Its low lignin content and high sugar content provide favorable conditions for highly efficient enzymatic processing and hydrolysis [48]. So far, sugar beet pulp has been mainly used as livestock feed, but recent publications present the possibility of its valorization in the production of industrially important biochemical compounds, which are discussed in the next subsection of the paper 

According to *The Cambridge Dictionary* [49], straw is the dry, stemmed plant material left over after harvesting crops such as wheat, barley, and oats. It has a wide range of uses in animal husbandry, in agriculture, as a soil mulching material, which helps retain moisture [50] and inhibits weed growth, as a mulch in gardens, and as an insulating material [51]. In addition, straw is often used in the production of biofuels, both in the form of briquettes and pellets, which can be burned to generate energy [52,53]. In some regions, straw can also be used as a raw material for paper production [54]. The chemical composition of straw varies depending on the type of grain (Table 2).

**Table 2:** Chemical composition of brewer’s spent grain, brewer’s spent hops, sugar beet pulp, and example chemical composition of straw according to origin.

Type	Component	Dry Mass Content Range/%	Reference
Brewer’s spent grain	Hemicellulose	19.2–41.9	[46]
	Cellulose	0.3–33	
	Proteins	14.2–31.0	
	Lignin	11.5–27.8	
	Starch	1.0–12.0	
	Lipids	3.0–10.6	
	Ash	1.1–4.6	
Brewer’s spent hops	Nitrogen free extract	40.0	[47]
	Crude fiber	23.0–26.0	
	Proteins	22.0–23.0	
	Ash	6.0–6.5	
	Lipids	4.5	
Sugar beet pulp	Hemicellulose	25–36	[55,56]
	Cellulose	20–25	
	Pectin	20–25	
	Proteins	10–15	
	Ash	3.7	
	Lignin	1–2	
	Lipids	1.4	
Sugarcane	Cellulose	41.3	[57]
	Hemicellulose	33.2	
	Lignin	17.3	
	Ash	2.8	
Wheat	Cellulose	31.2	[58]
	Hemicellulose	21.8	
	Lignin	22.8	
	Ash	8.7	
Rice	Cellulose	36.3	[59]
	Hemicellulose	20.7	
	Lignin	9.4	
Quinoa	Cellulose	31	[25]
	Hemicellulose	20.8	
	Lignin	20	
Rapeseed	Glucan	31.5	[60]
	Hemicellulose	17.4	
	Lignin	17.8	
	Ash	6.7	
	Hemicellulose	26.0	
	Lignin	12.2	

Potatoes are used in a variety of food product technologies. They are the raw material base in the production of products such as potato chips, food spirits, potato flours/starch, starch products, ready-to-eat meals, and stuffing. As a waste product, potato peel is discarded, creating many disposal problems and subsequent environmental burdens. This waste contains nutrients such as protein and carbohydrates (Table 3), so potato peel can be managed as a low-cost source of these nutrients in microbial culture. Potato peels obtained through hand peeling exhibit high levels of protein (17%) and starch (12%). Conversely, potato peels obtained through grating display higher concentrations of cellulose (21%) and lignin (19%). Disparities are also notable in terms of water content, with hand-peeled peels containing more water compared to those obtained through grating [61]. This discrepancy likely arises from the less meticulous nature of hand peeling, resulting in peels retaining more potato flesh. Consequently, this explains the substantial differences observed in starch, cellulose, or lignin content.

Biotechnological applications of the discussed wastes, along with other types of waste, are presented in Table 4, which provides a comprehensive summary of current valorization efforts in this field.

## 2. Utilization of Selected Lignocellulosic, Pectin, and Starch Wastes in the Biotechnology of Food Additives and Other Important Chemicals

The discussed wastes are the focus of research aimed at developing various strategies for their biotechnological management. Table 4 enumerates the biosynthetic products—such as cell biomass, enzymes, polyhydroxy alcohols, oligosaccharides, biopolymers, acids, and terpenes—discussed in the paper, all obtained using a range of agricultural byproducts including brewer’s spent grain, sugar beet pulp, exhausted sugar beet pulp, sugarcane bagasse, various types of straw (sugarcane, wheat, rice, quinoa, and rapeseed), oat bran, rice bran, corn cobs, corn stover, corn stalks, pineapple cores, birch, potato pulp waste, and peels (potato, sweet potato, papaya, pomegranate, lemon, mandarin, orange, pineapple, and kiwi).

### 2.1. Microbial Proteins

There are a limited number of recent articles addressing the efficiency of yeast biomass cultivation itself on substrates composed of lignocellulosic waste. The biomass may be a source of alternative proteins. This may be an important solution to balance the protein deficiency due to the growing population of humanity. Currently, research on the biotechnological utilization of lignocellulosic waste primarily focuses on the production of second-generation ethanol using genetically modified *Saccharomyces cerevisiae* yeasts, which efficiently produce ethanol from various feedstock sources. However, naturally, they cannot metabolize xylose, which is the main pentose sugar released during hemicellulose degradation, a component of lignocellulose [114]. To avoid the need for genetic modifications in *S. cerevisiae* yeast, non-conventional yeast strains are being sought that naturally possess the ability to process these substrates present in hydrolysates of lignocellulosic waste [115]. At the same time, these non-conventional strains must be resistant to chemical inhibitors present in such hydrolysates [116]. The waste biomass after ethanol production may be used for protein isolation.

Monteiro de Oliveira et al. [10] studied the growth of nine non-conventional yeast strains on diluted birch hemicellulose hydrolysate. They reported the highest biomass density in *C. parapsilosis* (current name *Candida melibiosica* according to https://www.mycobank.org/) culture after 26 h of cultivation and *K. marxianus* after 24 h. Drzymala et al. [88] used hydrolysates of rye straw, oat bran, and rye bran as substrates for the culture of *Y. lipolytica* yeast. The tested strain showed growth in all tested hydrolysates. The highest increase in yeast biomass cultured in the oat bran hydrolysate was over 9 g L^−1^ after 120 h, with a total biomass yield of 0.141 g g^−1^ and a total productivity of 0.078 g h^−1^. The authors concluded that the level of yeast biomass growth achieved is not significantly different from the production values of similar processes using other waste materials. Parchami et al. [63] used BSG to grow the filamentous fungi *N. intermedia* and *A. oryzae* to produce single-cell proteins (SCPs) of food and feed quality. Cultivating *A. oryzae* on all the examined substrates resulted in 7% to 40% more biomass compared to *N. intermedia*. Cultivating *A. oryzae* in organosolv liquor resulted in the highest biomass protein content (44.8 ± 0.7%) with a fungal biomass concentration of 5.1 g L^−1^. Patelski et al. [96] utilized potato pulp waste to produce feed yeast biomass. A comparison was made between two yeast strains, *Pichia stipitis* and *Candida guillermondii* (current names *Scheffersomyces stipitis* and *Meyerozyma guilliermondii*), and two hydrolysis methods for preparing culture media. The highest biomass yield (39.3% ― the biomass yield, expressed as the percentage of sugars converted into dry matter) after 48 h was achieved by *C. guilliermondii* yeast cultured on an enzyme hydrolysate-based medium. Thiviya et al. [106] utilized waste from pineapple (*Ananas comosus*), watermelon (*Citrullus lanatus*), papaya (*Carica papaya*), sour orange (*Citrus medica*), banana (*Musa acuminata*), and mango (*Mangifera indica*) peels to produce SCPs using palmyrah (*Borassus flabellifer*) toddy containing a natural mixed culture of yeast and bacteria in a liquid fermentation system. Biomass yields ranged from 5.3 ± 0.6 to 11.7 ± 0.8 g L^−1^, with the lowest biomass yield observed for watermelon peels and the highest for papaya peels (11.7 ± 0.81 g L^−1^). Papaya peels generated a significantly higher protein yield of 52.4 ± 0.4%. The optimal fermentation conditions for papaya waste were set at pH 5.0, 25 °C, and 24 h. The results led to the conclusion that papaya peel waste is a suitable substrate for the production of protein-rich cellular biomass using a natural palmyrah mixed culture.

### 2.2. Enzymes

Finding alternative, cost-effective methods for enzyme synthesis is crucial for the food industry. Such advancements contribute to reducing production costs, fostering the development of high-quality products, promoting sustainability, and enhancing access to modern technologies. These improvements enable the food sector to respond more efficiently to evolving consumer demands and environmental challenges.

The results of different studies present the possibility of utilizing brewer’s spent grain by using it as a raw material for enzyme synthesis. Hassan et al. [65] verified BSG as a substrate for the production of xylanopectinolytic enzymes in the culture of fungi isolated from spoiled fruits, including Bramley apples, Crimson grapes, Melody grapes, Tupi blackberries, and Imara raspberries. The highest enzymatic activity was achieved in the culture of *Mucor* sp. realized in a medium enriched with 1% xylan or pectin to initiate the synthesis reaction of xylanase or pectinase at 30 °C. Enzymatic activity was achieved with 137 U g^−1^ of BSG and 67 U g^−1^ of BSG for pectinase and xylanase, respectively. Llimós et al. [68] carried out a study on the synthesis of lignocellulosic enzymes (xylanases and cellulases) using brewer’s spent grain as a low-cost carbon source. In addition, after hydrolysis of BSG with the above-mentioned enzymes, the authors used the hydrolysate to produce biodegradable polymers—polyhydroxyalkanoates (PHAs); more information on this is available in the paragraph on biopolymers. Of the tested fungal strains, the molds *A. niger* and *T. aurantiacus* achieved the highest xylanase activities (about 268 and 241 U g^−1^ BSG, respectively). Faria et al. [64] obtained the highest xylanase activity in a culture of *M. aphidis* on medium pretreated with acid, reaching 518 U mL^−1^. In a study reported by Liguori et al. [67], *A. niger* was selected for its high capacity to produce cellulase and xylanase on BSG-based medium. Cellulase synthesis reached a maximum level after 10 days of fermentation of about 118 U g^−1^ of BSG, while maximum xylanase production was reached after 4 days with a result of about 1315 U g^−1^ of BSG. A similar study was conducted by Outeirino et al. [69]. The authors evaluated brewer’s spent grain as a substrate for the production of enzyme cocktails, mainly containing xylanase, cellulase, *β*-glucosidase, and ferulic acid esterase, in a fermentation process carried out by *A. brasiliensis*. Enzymatic activity measured after 4 days of fermentation was at about 3152 U g^−1^ of BSG, 7 U g^−1^ of BSG, 19 U g^−1^ of BSG, and 1 U g^−1^ of BSG, respectively, for xylanases, cellulases, *β*-glucosidases, and ferulic acid esterases, which are very promising results, especially that for xylanases. Interesting results were presented by Terrasan and Carmona [70], who determined the extent of the use of BSG for the synthesis of xylanolytic enzymes produced by *P. janczewskii*. The maximum observed activity of xylanase was 370 U g^−1^, *β*-xylosidase 247 mU g^−1^, and *α*-L-arabinofuranosidase 675 mU g^−1^. BSG hydrolysate was also used to compose media to produce thermostable α-amylase by *B. stearothermophilus*. The results indicated that the maximum synthesis of amylase was 198 U mL^−1^. This result was 1.3 times higher than that noted in the control medium (about 152.3 U mL^−1^). The obtained *α*-amylase showed antibiofilm activity against many pathogens [74]. Leite et al. [66] achieved the highest xylanase and cellulase activities (in the range of 300–313 U g^−1^ and 51–62 U g^−1^ for xylanase and cellulase, respectively) in the culture of *A. ibericus* strains on medium containing BSG as substrate. For β-glucosidase, the highest activity was achieved in *A*. *niger* cultures at 94 U g^−1^ also in BSG medium. In other studies, brewer’s spent grain was used as a culture medium for keratinolytic bacteria. *B. cereus* dominated in terms of proteolytic enzyme synthesis, while *B. subtilis* showed higher production of amylases, cellulases, and xylanases. Analysis of proteases showed that they were mainly enzymes with a molecular mass (*M*) > 70 kDa and a maximum activity of 2.49 U recorded after 5 days of culture in mineral-enriched medium. In contrast, maximum amylase activity was determined after 6 days of culture at 3.7 U [117]. BSG has been used as a substrate to produce the lignin-degrading enzyme laccase in the fermentation of *T. versicolor*. The increasing importance of laccase in biotechnology, pharmaceuticals, and the food industry has resulted in a growing demand for this enzyme. In response to this demand, an inexpensive raw material for synthesizing this protein is being sought. Tišma et al. [118] determined the maximum activity of lactase on the seventh day of solid fermentation with an average activity of 560 U L^−1^. The results indicate that the use of BSG as a fermentation raw material of white rot fungi may be a promising strategy to produce laccase. Almowallad et al. [71] used sugar beet pulp for the synthesis of endo-polygalacturonase by *A. niger*, *P. oxalicum,* and *P. variotii*. The highest endo-polygalacturonase activity was obtained in static fermentation of *A*. *niger* (at about 54%) and in shaken fermentation of *P*. *oxalicum*, obtaining an enzyme activity of about 53%, using a medium containing 3% sugar beet pulp in static cultures and 2% in shaken cultures. Wheat straw was also used for enzyme production. Shahryari et al. [84] evaluated the synthesis of phytase in the fermentation of wheat straw using the strain *A. ficuum*. Optimization of process parameters resulted in an increase in phytase activity from about 0.74 U g^−1^ to a maximum value of about 16.5 U g^−1^ of dry substrate. Ketsakhon et al. [85] used rice straw as a low-cost substrate to produce xylanase using the newly identified strain *B. altitudinis* and obtained xylanase with an activity of 2519 U mL^−1^. A similar study was conducted by Gautam et al. [73] using the same raw material in the synthesis of xylanase, but they used the white rot fungus strain *S. commune* for fermentation and found a maximum enzyme activity of 6722 U g^−1^ of dry substrate. In the study conducted by Matrawy et al. [83], xylanase activity of 54 U mL^−1^ was observed using wheat straw and *P. chrysogenum*. Campioni et al. [72] used sugarcane straw to produce xylanases and cellulases in immersion fermentation of *Trichoderma* and *Aspergillus*. The highest xylanase production (90 U mL^−1^) and the highest cellulase activity (0.5 FPU mL^−1^) were determined for *T. reesei* fermentation. Synthesis of cellulase from rice straw was carried out by Ismail and Hassan [86] in a solid fermentation of *A. terreus*. Cellulase activity was determined at 125 U g^−1^ of dry substrate. Mahmood et al. [97] conducted a solid potato peel fermentation process using *A. niger* to synthesize *α*-amylase. The optimal activity of the enzyme was determined to be 3014 U g^−1^. Similar studies were conducted by Mukherjee et al. [98], obtaining *α*-amylase with an activity of 1112 U g^−1^ of dry substrate, and Olakusehin and Oyedeji [99], who used *A. flavus* to produce α-amylase by solid-state fermentation, obtaining a maximum enzyme activity of 48 U mL^−1^. A different approach to enzyme synthesis from potato peels was presented by Niyomukiza et al. [100], who used *B. aerius* for the submerged fermentation process. With optimized culture conditions, the researchers obtained 17 U mL^−1^ of amylase and 12 U mL^−1^ of protease. Simultaneous synthesis of amylases and proteases was also studied by Tuysuz et al. [101]. The highest amylase and protease activities were determined at 65 U mL^−1^ and 26 U mL^−1^, respectively, using *A. rupiensis* and maintaining sterile process conditions. Potato peels have also been used as a substrate for phytase synthesis. Tian and Yuan [104] investigated the possibility of using solid-state fermentation and optimizing the conditions of the phytase synthesis process by *A. ficuum*. The highest enzyme activity, 13 U g^−1^, was obtained using (NH_4_)_2_SO_4_ as a nitrogen source. On the other hand, Ahmed et al. [102] used *T. purpureogenus* fungus in their study and obtained phytase with a maximum activity of 138 U mL^−1^. Waste from corn cobs was used to construct a substrate for xylanase production by Elegbede and Lateef [90]. The study was conducted using both submerged and solid-state fermentations with eight different fungal strains. In submerged fermentation, the highest xylanase activity reached approx. 51 U mL^−1^ after 168 h, whereas in SSF, the highest activity reached approx. 49 U g^−1^ after 120 h. It was shown that these enzymes improved bread dough rise by 1.87–2.20 times and increased orange juice clarity by 58–74%. Kumar et al. [89] used various agro-industrial wastes, i.e., potato peels, banana peels, orange peels, sawdust, pea peels, wheat bran, and rice bran, to produce laccase by submerged fermentation. Among all wastes, the maximum production of laccase (approx. 4.6 U mL^−1^) was achieved using rice bran. Lemon peels were used by Gooruee et al. [110] for the production of extracellular enzymes by various *Trichoderma* fungal species in submerged fermentation. The maximum activities of all cellulolytic enzymes, xylanase, and pectinase were determined to occur at about 11 U mL^−1^, 4 U mL^−1^, and 2 U mL^−1^, respectively. Pomegranate peels were proposed by Atalla and Gamal [107] to synthesize xylanase using *C. globosum*. The results showed that as the concentration of waste increased (up to a content of 40 g L^−1^), xylanase activity increased, after which it decreased. Xylanase activity at the optimal waste concentration was determined at a maximum level of approx. 1400 U mL^−1^. In addition, it was observed that the addition of calcium chloride increased xylanase activity to approx. 1470 U mL^−1^.

The above review shows how much progress has already been made toward the use of plant-derived wastes for synthesizing enzymes with diverse properties, like amylase, cellulase, endoglucanase, endo-polygalacturonase, exoglucanase, ferulic acid esterases, laccase, pectinase, phytase, protease, xylanase, α-amylase, α-L-arabinofuranosidase, β-glucosidase, and β-xylosidase. This is the most popular current trend in valorizing lignocellulosic, pectin, and starch wastes.

### 2.3. Polyhydroxy Alcohols

The growing problem of obesity, diabetes, and dental caries is motivating the food industry to seek low-calorie sugar substitutes with a low glycemic index [119]. Polyhydroxy alcohols offer an alternative to sucrose as sweeteners. As a result of the growing interest and demand for sweeteners, low-cost methods of producing these substances are being sought, and these include polyhydroxy alcohols such as xylitol and arabitol.

Different lignocellulosic materials in the culture of *K. pastoris* yeast cells were studied for xylitol biosynthesis [75]. The highest xylitol production of about 1.5 g L^−1^ was achieved in BSG hydrolysate. In a further study, the BSG hydrolysate was detoxified using activated carbon to remove growth inhibitors such as HMF, furfurals, and acetic acid. This resulted in an improvement in xylitol synthesis to a level of about 4 g L^−1^. In addition, a process of decreasing arabinose content was noted, and with it the synthesis of arabitol, to a maximum concentration of 0.8 g L^−1^. These results justify the use of lignocellulosic wastes, especially brewer’s spent grain, as a raw material for the synthesis of xylitol and arabitol in *K*. *pastoris* culture. Singh et al. [87] conducted a study on the pretreatment of rice straw used for xylitol production. They obtained the highest xylitol yield of 26 g L^−1^, with a fermentation efficiency of 63% and productivity of 0.26 g h^−1^ L^−1^, using strain *C. tropicalis* after a 96 h process carried out at 30 °C. On the other hand, Jin et al. [25] conducted a study on the synthesis of xylitol from quinoa straw hydrolysate subjected to a two-step detoxification process (activated carbon adsorption and vacuum evaporation). After 96 h of fermentation, the xylitol yield reached 0.5 g g^−1^. According to the authors, this was 1.2 times higher compared to the yield obtained in the fermentation of the hydrolysate subjected only to vacuum evaporation. López-Linares et al. [60] evaluated the feasibility of using rapeseed straw hydrolysate as a fermentation medium for xylitol synthesis. Results of 0.55 g g^−1^ and 0.45 g g^−1^ were obtained for *C*. *guilliermondii* and *D*. *hansenii*, respectively. Barathikannan et al. [109] enhanced the parameters for maximizing yields, including xylitol, through the utilization of various substrates subjected to acid–base hydrolysis. Employing pomegranate peel as a substrate resulted in the highest xylitol yield of 56 g L^−1^ after 60 h of cultivation. Nasoha et al. [112] evaluated pineapple peel hydrolysate as a sustainable carbon source for xylitol production. Fermentation process tests with *C. tropicalis* showed comparable performance of the hydrolysate to a control medium containing acetic acid. The implication is that the fermentation process remains undisturbed by the natural antioxidants found in pineapple peel, and that the inhibitory effects are mainly related to acetic acid. Specifically, the elevated pH level was found to mitigate the effects of acetic acid during fermentation, leading to increased xylitol production. In addition, the study authors used urea as a nitrogen source, which proved to be a cost-effective substitute for yeast extract, yielding a commendable overall xylitol yield of 0.3 g g^−1^. The study by Mardawati et al. [95] focuses on the production of xylitol from waste from the pineapple industry. Pineapple cores were used as the raw material, subjecting them to enzymatic and acid hydrolysis processes. The results showed a high content of hemicellulose (36.06%), suggesting its potential for xylitol production. The efficiency of enzymatic hydrolysis was found to be higher than that of acid hydrolysis, and the highest xylitol and biomass yields were achieved using *C. tropicalis* and enzymatic hydrolysate, reaching results of 0.4 g of xylitol per g of glucose and 0.2 g of biomass per g of glucose. Despite the increasing interest in alternative sugar sources and substitutes, there is limited recent information regarding the utilization of pectin waste for producing polyhydroxy alcohols. In the field of sweetener synthesis, work was mainly carried out using waste rich in xylans. However, pectin waste could also be used in this area.

### 2.4. Oligosaccharides

The beneficial effects of different oligosaccharides on human and animal health, including through the prevention of gastrointestinal disorders, allows them to qualify as prebiotics. Additionally, they exhibit acceptable organoleptic properties [120], which can be used in the formulation of functional foods. Examples of oligosaccharides are xyloligosaccharides (XOSs), which can be obtained from lignocellulosic biomass. Amorim et al. [76] presented the results of a study to optimize the release, by hydrolysis, of XOSs from waste through direct fermentation using two species of *Trichoderma* fungi. The best results were obtained during fermentation on a brewer’s spent grain substrate. The highest production yield of XOSs was achieved at 38 mg g^−1^ (xylose equivalent) under optimal conditions, i.e., fermentation conducted for 3 days at pH 7.0 at 30 °C and the presence of 20 g of BSG per L in the culture medium. A better yield of arabino-xyloligosaccharides (AXOSs) was obtained by Amorim et al. [77]. *B. subtilis* subjected to genetic recombination was used for the fermentation process, running for 12 h at pH 7.0 and 45 °C in medium with a load of 20 g of BSG per L. Xylose concentration was determined at 54 mg g^−1^ (xylose equivalent), signaling a 33% increase in process efficiency compared to the xylose released by a wild-type strain of this species (40.67 ± 1.65 mg g^−1^). The above results highlight the potential of using BSG waste in the fermentation of a functional food ingredient with prebiotic properties. However, it is necessary to optimize the process in order to minimize the synthesis of inhibitors, thus increasing the efficiency of the microbial hydrolysis process of the waste. In addition, the chemical structure and prebiotic activity of the obtained xyloligosaccharides should be characterized. The Yang et al. [111] study aimed to harness the biomass from citrus peel wastes through fermenting using the engineered yeast strain *P. pastoris* (current name *Komagataella pastoris)*. The results demonstrated that fermentation of the engineered yeast strain could yield significant quantities of oligogalacturonides with a degree of polymerization ranging from 2 to 7. The oligogalacturonide content measured at 4.5 and 2 mg mL^−1^ corresponded to oligosaccharide yields of 26.1% and 15.7% for mandarin and orange peel substrates, respectively.

The literature from recent years contains a limited amount of readily accessible data regarding the utilization of the discussed plant-derived wastes in microbiological cultivation aiming to promote oligosaccharide release by hydrolysis. This scarcity highlights a significant research gap in the field, indicating an area where further investigation and exploration are warranted. Closing this gap through targeted research efforts could not only contribute to expanding our understanding of sustainable resource utilization but also unlock potential innovations with practical implications across multiple industries.

### 2.5. Biopolymers

β-Glucan, a polysaccharide found in the cells of bacteria, fungi, and yeast, assumes a pivotal role in biotechnological applications owing to its distinctive properties. Its ability to stimulate the immune system makes it a potential future ingredient in dietary supplements and pharmaceuticals [121,122,123]. Moreover, β-glucan demonstrates prebiotic attributes, fostering the proliferation of beneficial bacteria within the digestive tract [124]. Furthermore, β-glucan exhibits promise in the food industry as a functional additive for enhancing product texture and stability [125]. It is worth noting that the structure of β-glucan determines its properties [126]. Variations in this compound’s structure can result in alterations in its biological activity and its ability to interact with other substances [127]. Hence, comprehending the structure of β-glucan is crucial for elucidating its biological functions and exploring its potential applications across various domains, including medicine, nutrition, and cosmetology. β-Glucans, as multifunctional biopolymers, are commonly produced by microorganisms on substrates containing commercial sugars, which is not an economical solution. Abdeshahian et al. [57] explored the feasibility of converting sugarcane straw (SCS), an agricultural residue, into β-glucan using the fungal strain *L. theobromae*. To enhance sugar recovery, SCS underwent a two-stage pretreatment: initial exposure to dilute sulfuric acid (1% *w*/*v*) to disrupt hemicellulose, followed by alkaline treatment (1% NaOH) to solubilize lignin. The resulting cellulose-rich substrate was enzymatically broken down, yielding a glucose solution (48.65 g L^−1^) suitable for fermentation. During batch cultivation, optimal β-glucan synthesis occurred at pH 7 and a low yeast extract concentration (1 g L^−1^). After 72 h, the process achieved a product yield of 4.7% (relative to glucose consumed) and a volumetric productivity of 0.014 g L^−1^h^−1^. The researchers proposed that nitrogen scarcity in the medium triggered a stress response in the fungus, redirecting metabolic activity toward β-glucan biosynthesis. Ascencio et al. [82] investigated the potential use of sugarcane bagasse (SCB) and rice/soybean bran for the production of lasiodiplodan, a β-(1 → 6)-D-glucan-type exopolysaccharide. The substrate preparation process involved sequential chemical treatment (nitric acid → NaOH), followed by enzymatic hydrolysis using cellulases. Optimization of the cultivation conditions for *L. theobromae* in a batch system enabled the achievement of a record lasiodiplodan concentration of 22 g L^−1^ using SCB and rice bran as a nitrogen source. A review of articles on β-glucan production using plant waste as a culture medium reveals an important knowledge gap. To date, a small number of readily available articles have been published addressing this topic. Despite the promising results presented above, further research and experimentation involving a wider range of plant materials are needed. The small number of plant wastes studied suggests that this topic is still under-researched and requires more attention from researchers. Further research should include a broader range of plant wastes, as well as more comprehensive analyses of their potential as substrates for β-glucan production. This approach will enable a better understanding and utilization of this methodology in the industry.

Bacterial cellulose has attracted considerable interest due to its unique physicochemical properties compared to plant cellulose. It represents an extremely pure and natural exopolysaccharide. Among other things, it exhibits the ability to protect against UV radiation, maintain optimal oxygen levels, and retain moisture, making it an ideal dressing material. Bacterial cellulose is currently of interest to the food industry for use in packaging production [128,129,130,131]. Bacterial cellulose (BC) was biosynthesized using agricultural waste, specifically potato peel hydrolysate, as a sustainable nutrient source. The fermentation process, conducted with *G. xylinus* under optimized conditions (35 °C, 6 days), achieved a maximum BC yield of 4.7 g L^−1^. Analytical characterization revealed that the synthesized BC exhibited enhanced crystallinity (82.5%), uniform particle distribution (PDI 0.195), and a highly ordered fibrillar network [103]. Güzel and Akpınar [113] explored the valorization of agro-industrial waste by converting peels from seven fruits and vegetables (cucumber, melon, kiwi, tomato, apple, quince, and pomegranate) into bacterial cellulose (BC) using *K. hansenii*. The peels underwent acid hydrolysis (0.6 M H₂SO₄), and the resulting hydrolysates were fermented. Notably, pomegranate peel hydrolysate failed to support BC synthesis, likely due to inhibitory phenolic compounds. Among successful substrates, kiwi peel yielded the highest BC production (11.5%), whereas apple peel generated the lowest (1.5%). The study highlights the potential of fruit/vegetable waste as a low-cost carbon source for BC biosynthesis, aligning with circular economy principles. In addition, bacterial cellulose from kiwi peels had the highest crystallinity and thinnest fibers. The work of Cheng et al. [94] addressed the synthesis of bacterial cellulose in *A. xylinum* culture in corn stalk hydrolysate as a carbon source. Corn stalk underwent acetic acid pretreatment, followed by inhibitor removal via sequential adsorption (activated carbon) and ion exchange resin treatments. The integrated detoxification strategy significantly enhanced BC synthesis by removing inhibitors like furfural and lignin, achieving a yield of 3 g L^−1^. Microscopic imaging (SEM) revealed nanoscale BC fibrils, with diameters spanning 20–70 nm.

Like bacterial cellulose, polyhydroxyalkanoates find application in the production of food packaging and protective coatings [132]. Llimós et al. [68], having investigated the synthesis of lignocellulosic enzymes (xylanases and cellulases) utilizing brewer’s spent grain, proceeded to utilize the hydrolysate for the production of biodegradable polymers known as polyhydroxyalkanoates (PHAs). The enzyme hydrolysate, derived from *A. niger*, was employed in the synthesis of PHAs using two bacterial strains. The optimal yield was achieved utilizing *C. necator* after 48 h of cultivation, yielding 9 mg of PHA per gram of BSG.

### 2.6. Carboxylic Acids

Organic acids play a crucial role in food technology, serving diverse functions including preservation, pH regulation, and enhancement of food product quality and shelf life. Their natural origins and efficacy in suppressing microbial growth contribute to their rising popularity as substitutes for synthetic preservatives [133,134]. Additionally, organic acids are frequently employed as feed additives owing to their beneficial impact on animal health and performance. They possess the capability to facilitate digestion, enhance intestinal health, and inhibit pathogen proliferation, collectively fostering improved animal welfare and productivity [135,136].

Citric acid is used in various industries, including food, pharmaceuticals, cosmetics, and chemicals. It has many important functions, including as an acidifier, antioxidant, preservative, or flavor enhancer. In addition, recent publications report the possibility of using citric acid to crosslink biopolymer films used in food packaging [78]. Many microorganisms have the ability to synthesize citric acid, but it is *Aspergillus niger* that is the organism used in the industrial biosynthesis of this compound, due to the highest efficiency of its synthesis [108]. Pathania et al. [108] conducted a study on citric acid production using BSG as the raw material. The authors tested the effect of different supplements on fermentation yield. The results showed that the addition of 0.1% peptone increased citric acid production from 0.15% to 0.19% compared to yeast extract, the addition of which lowered the synthesis yield. The addition of potassium dihydrogen phosphate at an amount of 0.1% contributed to the highest citric acid synthesis yield of 0.18%. The minerals ammonium sulfate and magnesium sulfate also showed the highest efficiency at a concentration of 0.1%. The researchers concluded that brewer’s spent grain can be a low-cost raw material in the production of citric acid, but this will only be possible with a properly composed medium with appropriate amounts of organic compounds adapted to the strain used. Citric acid production from dried and undried pomegranate peel waste by *A. niger* fungi in SSF under non-aseptic conditions was investigated by Roukas and Kotzekidou [105]. The results show that the concentration of citric acid produced from undried pomegranate peels was higher compared to that obtained from pomegranate peels dried at 45 °C for 48 h or at 105 °C for 4 h. The authors obtained 307 g kg^−1^ of dry peel citric acid after 8 days of fermentation at 25 °C using undried pomegranate peel waste. Moreover, the addition of methanol at a concentration of 3% (*w*/*w*) to the undried waste promoted citric acid biosynthesis, resulting in a significant increase in citric acid production. To produce citric acid using *A. niger* through submerged fermentation, sweet potato peel waste served as the primary carbon source. By optimizing parameters including carbon source (97.25%), nitrogen concentration (1.25% *w*/*v*), fermentation time (7 days), and pH (6.5), citric acid production increased by 135%, resulting in a yield of 4.4 mg mL^−1^ [137].

Lactic acid has received more attention in recent years, due to its role in the production of polylactic acid (PLA), as a safe, biodegradable, and biocompatible polymer. Polylactic acid (PLA) is used to produce biodegradable plastics that have environmental applications [138] and is being investigated as an addition to eco-friendly food packaging [139]. In food production, lactic acid/polylactic acid is used as a preservative and packaging material, among other things [140,141]. Díaz et al. [79] carried out lactic acid production with sugar beet pulp using the SSF method. A maximum lactic acid concentration of 27 g L^−1^ with a yield of (13 ± 1)% and a maximum productivity of (0.63 ± 0.01) g L^−1^ was obtained in SSF-type culture with controlled pH and nutrient supplementation by adding 20 vol.% of MRS medium. Marzo et al. [80] determined lactic acid production using sugar beet pulp subjected to different types of pretreatments. Each type of pretreatment increased the relative cellulose content by breaking down pectins and hemicellulases. The best results were obtained by subjecting the sugar beet pulp to thermochemical treatment with 1% *w*/*v* sulfuric acid, followed by fermentation with *L. plantarum*. In such an experimental setup, the authors obtained lactic acid at a level of 50 g L^−1^ (in an experiment using untreated beet pulp, lactic acid was determined to be 27 g L^−1^). A similar study was conducted by Marzo et al. [81], who valorized exhausted sugar beet pulp (ESBPP) as a raw material for lactic acid fermentation. The researchers subjected the waste to enzymatic hydrolysis using *Aspergillus awamori* 2B.361 U2/1, and then composed a medium that was used for lactic acid production. The highest concentration of 30 g L^−1^ was obtained from fermentation of the hydrolysate enriched with yeast extract (5 g L^−1^) and with pH control with CaCO_3_, using *L. plantarum*. Marzo et al. [81] indicated that the efficiency of lactic fermentation can be improved by supplementing the hydrolysate with yeast extract and controlling the pH during the process. When comparing the results obtained to those of Marzo et al. [80], the fermentation yield is much lower.

### 2.7. Terpenes

Terpenes are a group of chemical compounds found in plants that have been used in traditional medicine for centuries. Some of these compounds exhibit a wide range of therapeutic properties, such as antibacterial, antiviral, anticancer, or anti-inflammatory effects, making them valuable in biomedical research and clinical practice. In addition, these compounds are safe due to their very low toxicity [142,143]. Terpenes combine technological functions with sensory and health benefits, making them a crucial component of innovative food products. The growing popularity of natural ingredients further supports their expanded use across various branches of the food industry [144]. One of the few articles addressing the synthesis of terpenes in substrates composed of wastes of plant origin was written by Yao et al. [91]. The authors touched on preliminary studies on engineering the oleaginous yeast *Y. lipolytica* to enhance limonene production using xylose hydrolysate and lignocellulose. As reported by the authors, the production of limonene reached maximum production at 21 mg L^−1^ after 72 h using 50% detoxified corn stover hydrolysate. The authors claim that this study is one of the first to describe the production of limonene by yeast from lignocellulosic raw materials (to the best of their current knowledge). Bi et al. [92] explored the potential of utilizing a genetically modified strain of *Y. lipolytica* for β-farnesene production using corn stover hydrolysate as the primary carbon source. Through a combination of metabolic engineering strategies, along with the utilization of lignocellulosic hydrolysate and the addition of Mg^2+^, β-farnesene production was promoted. The highest yield of β-farnesene, reaching 7 g L^−1^, was achieved after 144 h of fermentation using lignocellulosic hydrolysate. Kirby et al. [93] optimized the production of monoterpene 1,8-cineole and sesquiterpene α-bisabolene using *R. toruloides* in a medium containing corn hydrolysate. Maximum terpene concentrations were determined at 2.6 g L^−1^ and 1.4 g L^−1^ with yields of 0.022 g and 0.012 g of terpene per 1 g of sugar for α-bisabolene and 1,8-cineole, respectively. The authors indicate the necessity of reducing the pathway of lipid synthesis from acetyl-CoA (lipogenesis) to increase the efficiency of synthesis of the compounds in question. An underexplored aspect in the literature is the topic of terpene synthesis by microorganisms on ecological medium composed using plant waste. Further exploration is warranted to investigate the optimization of terpene synthesis pathways in microorganisms utilizing ecological mediums derived from plant waste. This could involve conducting systematic studies to identify key factors influencing terpene production, such as nutrient composition, pH levels, and fermentation conditions. Additionally, there is a need for comprehensive research into the potential scalability and economic feasibility of terpene production from plant waste-derived mediums. This could include evaluating the cost-effectiveness and sustainability of large-scale production processes, as well as assessing the potential environmental impacts and regulatory considerations.

## 3. Conclusions

In recent years, significant progress has been made in understanding the potential of plant-origin agricultural and food processing wastes as a valuable feedstock for the biotechnology industry. Nevertheless, we cannot stop there, as our planet faces increasingly serious challenges of environmental degradation. In terms of our future, continuing research into the use of plant-origin waste is of great importance. Not only does it help reduce the environmental burden of industrial waste, but it also opens up new economic prospects and contributes to achieving the sustainable development goals under the European Green Deal. Summarizing the literature review, significant progress has been made in recent years in understanding the potential of utilizing plant-based wastes as valuable raw materials for the biotechnology industry. Research in this area has primarily focused on enzyme production. The findings from these studies are promising, indicating the potential for efficient, environmentally friendly, and cost-effective production. However, there are areas that necessitate further research. These include exploring the feasibility of utilizing other types of plant waste, such as brewer’s spent hops, for which there is a lack of literature. Additionally, there is a gap in the research concerning the utilization of waste in microbial culture for the production of terpenes, oligosaccharides, and biopolymers. In the context of the efficient utilization of plant-based wastes in microbial cultures, the necessity for pretreatment of these wastes presents significant limitations, such as costs associated with enzymes and equipment, as well as the use of chemicals, which may raise ecological concerns. Furthermore, there is a need for more comprehensive research on the potential applications of waste-derived substances in the biotechnology industry, along with an assessment of their performance and economic viability on a large scale. Therefore, it is worthwhile in the future to carry out detailed economic analyses assessing the viability of technologies using lignocellulosic, pectin, or starch wastes, especially in the context of regional variations in the cost of raw materials. There is still a lot of lignocellulosic, pectin, and starch waste that needs to be examined for its potential use as a raw material in microbial culture, and the spectrum for obtaining compounds important in biotechnology is very wide. It is worthwhile to take an interest in the production of yeast biomass with therapeutic properties, e.g., biomass rich in immunoactive polysaccharides, which may, in the future, contribute to minimizing the use of antibiotics in animal production. In this respect, there are no literature data regarding the use of the discussed lignocellulosic, pectin, and starch wastes or other types of wastes. Another suggested research direction involves exploring unconventional strains of microorganisms that can efficiently process specific types of waste while demonstrating resistance to inhibitors generated during pretreatment. As technologies and our knowledge develop, we will be able to convert lignocellulose more efficiently into simple substances desirable for biotechnology and industry while contributing to the protection of our planet.

## Figures and Tables

**Table 1:** A representation of the number of records retrieved from the PubMed database for literature queries related to the major wastes reviewed in this article (https://pubmed.ncbi.nlm.nih.gov, accessed 3 February 2024, modified 20 February 2025).

Literature Query	Number of Results	Literature Query	Number of Results	Literature Query	Number of Results
Lignocellulose	15,032	Lignocellulosic waste	2776	Biotechnological use of lignocellulose	606
Brewer’s spent hops	5	Brewer’s spent hops waste	2	Biotechnological use of brewer’s spent hops	No results
Brewer’s spent grain	402	Brewer’s spent grain wastes	163	Biotechnological use of brewer’s spent grain	16
Straw	16,620	Straw waste	2410	Biotechnological use of straw	111
Wheat straw	4658	Wheat straw waste	849	Biotechnological use of wheat straw	64
Rice straw	4529	Rice straw waste	987	Biotechnological use of rice straw	28
Quinoa straw	18	Quinoa straw waste	3	Biotechnological use of quinoa straw	No results
Rapeseed straw	188	Rapeseed straw waste	36	Biotechnological use of rapeseed straw	1
Sugarcane straw	398	Sugarcane straw waste	126	Biotechnological use of sugarcane straw	12
Sugarcane bagasse	2404	Sugarcane bagasse waste	630	Biotechnological use of sugarcane bagasse	89
Oat bran	655	Oat bran waste	13	Biotechnological use of oat bran	2
Rice bran	3104	Rice bran waste	250	Biotechnological use of rice bran	17
Corn cob	535	Corn cob waste	158	Biotechnological use of corn cob	10
Corn stover	2011	Corn stover waste	301	Biotechnological use of corn stover	16
Corn stalk	1524	Corn stalk waste	202	Biotechnological use of corn stalk	8
Pineapple cores	8	Pineapple core waste	2	Biotechnological use of pineapple cores	No results
Starch	86,431	Starch wastes	1859	Biotechnological use of starch	423
Potato peels	321	Potato peel wastes	101	Biotechnological use of potato peels	4
Potato pulp	203	Potato pulp waste	36	Biotechnological use of Potato pulp	1
Sweet potato peel	52	Sweet potato peel waste	13	Biotechnological use of sweet potato peel	No results
Pectin	14,351	Pectin waste	576	Biotechnological use of pectin	141
Sugar beet pulp	691	Sugar beet pulp waste	110	Biotechnological use of sugar beet pulp	15
Papaya peels	49	Papaya peel waste	19	Biotechnological use of papaya peels	No results
Pomegranate peel	904	Pomegranate peel waste	155	Biotechnological use of pomegranate peel	2
Lemon peel	2345	Lemon peel waste	456	Biotechnological use of lemon peel	13
Mandarin peel	215	Mandarin peel waste	36	Biotechnological use of mandarin peel	1
Orange peel	1487	Orange peel waste	378	Biotechnological use of orange peel	8
Pineapple peel	183	Pineapple peel waste	83	Biotechnological use of pineapple peel	No results
Kiwi peel	27	Kiwi peel waste	11	Biotechnological use of kiwi peel	1

**Table 3:** The chemical composition of potato peels.

Component	Dry Mass Content Range/%		
	Raw Potato Peel *Solanum tuberosum* Variety Norchip [62]		Dried Potato Peel [61]
	Hand Peeled	Peeled by Grating	Peeled by Abrasion
Cellulose	8.0	21.0	8.3
Hemicellulose	―	―	7.41
Lignin	6.2	19.0	32.88
Protein	17.1	10.5	10.73
Starch	12.0	9.0	23.01
Ash	8.4	5.0	7.45
Water	4.9	2.5	―
Lipids	0	0	2.45

**Table 4:** Summary of products obtained as a result of biotechnological management of selected lignocellulosic wastes. (Latin names of microorganisms are included, used in accordance with the source data. For some microorganisms, new taxonomic names are in force, as indicated in the text of the work).

Waste	Group of Compounds	Products	Productivity	Composition of the Medium	Microorganism	Reference
Brewer’s spent grain (BSG)	Cell biomass/single-cell protein	Fungal biomass concentration	5.1 g L^−1^	Organosolv liquor with dry matter content 9.4 g L^−1^ and with composition (dry mass/%) of total lignin 5.06 ± 0.05; glucan 37 ± 2; xylan 5.7 ± 0.3; galactan 0.90 ± 0.08; arabinan 0.89 ± 0.07; protein 26.5 ± 0.7	*Aspergillus oryzae* var. *oryzae* CBS 819.72	[63]
		Single-cell protein (SCP)	44.8 ± 0.7%			
	Enzymes	Xylanase	518.2 U mL^−1^	[g L^−1^]: carbohydrates 40 (including 4% [*w*/*v*] d-xylose and beechwood xylan; 11% [*w*/*v*] BSG); MgSO_4_ 0.3; KH_2_PO_4_ 0.3; yeast extract 1	*Moesziomyces aphidis* PYCC 5535	[64]
			67 U g^−1^	The basic medium [g L^−1^]: KH_2_PO_4_ 2.0; (NH_4_)_2_SO_4_ 1.4; urea 0.3; MgSO_4_⋅7H_2_O 0.3; CaCl_2_ 0.3; [mg L^−1^]: FeSO_4_⋅7H_2_O 5.0; MnSO_4_⋅H_2_O 1.56; ZnSO_4_⋅7H_2_O 1.4; CoCl_2_ 2.0; BSG 15 g/250 mL; pH 6	*Mucor* sp. (AB1)	[65]
			300–313 U g^−1^	2 g of dry substrate; solid medium with moisture level adjusted to 75% (wet basis) with distilled water and ratio C/N fixed to 15 (value of BSG)	*Aspergillus ibericus*	[66]
			1315.15 ± 37.5 U g^−1^	25 g of dry BSG/250 mL solution moisture adjusted to 70% by adding enough volume of distilled water and a mineral salt solution containing [g L^−1^] KH_2_PO_4_ 1.5; CuSO_4_ 0.4; CoSO_4_ 0.0012	*Aspergillus niger* LPB-334	[67]
			268 ± 24 U g^−1^	Preparation of BSG for solid-state fermentation: adding 2 × 2 cm sponge pieces as a bulking agent (3% *w*/*w*) then distilled water to reach 78 ± 2% moisture content (MC)	*Aspergillus niger* ATCC 16888	[68]
			241 ± 10 U g^−1^		*Thermoascus aurantiacus* ATCC 26904	
			3152.39 ± 20.88 U g^−1^	400 g BSG (dry weight) moistened with a mineral salts solution containing [g L^−1^] 1.3 (NH_4_)_2_SO_4_; 5.0 NaNO_3_; 4.5 KH_2_PO_4_; 3 yeast extract in the proportion 1:2.5 [*w*/*v*]	*Aspergillus brasiliensis* CECT 2700	[69]
			370.0 ± 30.1 U g^−1^	5 g dried BSG; 50% initial moisture, provided by Vogel’s salt solution	*Penicillium janczewskii* CRM 1348	[70]
		Pectinase	137 U g^−1^	The basic medium [g L^−1^]: KH_2_PO_4_ 2.0; (NH_4_)_2_SO_4_ 1.4; urea 0.3; MgSO_4_⋅7H_2_O 0.3; CaCl_2_ 0.3; [mg L^−1^]: FeSO_4_⋅7H_2_O 5.0; MnSO_4_⋅H_2_O 1.56; ZnSO_4_⋅7H_2_O 1.4; CoCl_2_ 2.0; BSG 15 g/250 mL; pH 6	*Mucor* sp. (AB1)	[65]
		Cellulase	51–62 U g^−1^	2 g of dry substrate; solid medium with moisture level adjusted to 75% (wet basis) with distilled water and ratio C/N fixed to 15 (value of BSG)	*Aspergillus ibericus*	[66]
			118.04 ± 8.4 U g^−1^	25 g of dry BSG/250 mL solution; moisture adjusted to 70% by adding enough volume of distilled water and a mineral salt solution containing [g L^−1^] KH_2_PO_4_ 1.5; CuSO_4_ 0.4; CoSO_4_ 0.0012	*Aspergillus niger* LPB-334	[67]
			7.26 ± 0.13 U g^−1^	400 g BSG (dry weight) moistened with a mineral salt solution containing [g L^−1^] 1.3 (NH_4_)_2_SO_4_; 5.0 NaNO_3_; 4.5 KH_2_PO_4_; 3 yeast extract in the proportion 1:2.5 [*w*/*v*]	*Aspergillus brasiliensis* CECT 2700	[71]
		Laccase	560 U L^−1^	A 50 g aliquot of BSG, particle size 2–3 mm, mixed with 10 mL of distilled water	*Trametes versicolor* TV-6	[72]
		β-Glucosidase	94 ± 4 U g^−1^	2 g of dry substrate; solid medium with moisture level adjusted to 75% (wet basis) with distilled water and ratio C/N fixed to 15 (value of BSG)	*Aspergillus niger* CECT 2088	[73]
			19.02 ± 0.04 U g^−1^	400 g BSG (dry weight) moistened with a mineral salt solution containing [g L^−1^] 1.3 (NH_4_)_2_SO_4_; 5.0 NaNO_3_; 4.5 KH_2_PO_4_; 3 yeast extract in the proportion 1:2.5 [*w*/*v*]	*Aspergillus brasiliensis* CECT 2700	[69]
		β-Xylosidase	246.5 ± 14.7 mU g^−1^	5 g dried BSG; 50% initial moisture provided by Vogel’s salt solution	*Penicillium janczewskii* CRM 1348	[70]
		α-Amylase	198.09 U mL^−1^	[% *w*/*v*] starch 0.2; peptone 0.2; KCl⋅4H_2_O 0.02; MgSO_4_·7H_2_O 0.01; BSG hydrolysate (0.22% *v*/*v*)	*Bacillus stearothermophilus* LZT020	[74]
		Ferulic acid esterase	1.05 ± 0.06 U g^−1^	400 g BSG (dry weight) moistened with a mineral salt solution containing [g L^−1^] 1.3 (NH_4_)_2_SO_4_; 5.0 NaNO_3_; 4.5 KH_2_PO_4_; 3 yeast extract in the proportion 1:2.5 [*w*/*v*]	*Aspergillus brasiliensis* CECT 2700	[69]
		α-L-Arabinofuranosidase	674.8 ± 30.9 mU g^−1^	5 g dried BSG; 50% initial moisture provided by Vogel’s salt solution	*Penicillium janczewskii* CRM 1348	[70]
	Polyhydroxy alcohols	Xylitol	3.97 ± 0.10 g L^−1^	Detoxified BSG hydrolysates ([g L^−1^]: glucose 9.57 ± 1.76; xylose 13.00 ± 4.74; arabinose 8.85 ± 2.55) were used as cultivation medium supplemented with ammonium sulfate (10 mL, 13.6 g L^−1^)	*Komagataella pastoris* DSM 70877	[75]
		Arabitol	0.82 ± 0.05 g L^−1^			
	Oligosaccharides	Arabino-xylooligosacharide (AXOS)	38.3 ± 1.8 mg g^−1^	20 g L^−1^ BSG in 2% [*w*/*v*] Vogel media; pH 7.0	*Trichoderma reesei* MUM 9753	[76]
			54.24 ± 1.10 mg g^−1^		recombinant *Bacillus subtilis* 3610	[77]
	Biopolymers	Polyhydroxyalkanoate (PHA)	9.0 ± 0.44 mg g^−1^	20 mL hydrolysates BSG; [g L^−1^] 1 (NH_4_)_2_SO_4_; 1.5 KH_2_PO_4_; 9.02 Na_2_HPO_4_⋅12H_2_O; 0.1 CaCl_2_⋅2H_2_O; 0.2 MgSO_4_⋅7H_2_O; 1 mL L^−1^ of microelement solution [g L^−1^]: 0.1 ZnSO_4_⋅7H_2_O; 0.03 MnCl_2_⋅4H_2_O; 0.3 H_3_BO_3_; 0.2 CoCl_2_; 0.02 CuSO_4_⋅7H_2_O; 0.02 NiCl_2_⋅6H_2_O; 0.03 Na_2_MoO_4_⋅2H_2_O	*Cupriavidus necator* DSM428	[68]
	Carboxylic acids	Citric acid	0.12–0.23 g 100 g^−1^	150 mL of fermentation medium [g L^−1^]: peptone 2; yeast extract 1.5: potassium dihydrogen phosphate 2; magnesium sulfate 2; ammonium sulphate 2; brewer’s spent grain 30 g, containing water in 1:5 ratio; pH 5.5	*Aspergillus niger* MTCC 281	[78]
Sugar beet pulp (SBP)	Enzymes	Endo-polygalacturonase	54.44 ± 1.4%	[g L^−1^]: SBP powder 30; (NH_4_)_2_HPO_4_ 2; NH_4_H_2_PO_4_ 0.9; MgSO_4_ 0.1; KCl 0.5; pH 5.6 and 7.0	*Aspergillus niger* AUMC 4156	[71]
			52.94 ± 2.0%		*Penicillium oxalicum* AUMC 4153	
Exhausted sugar beet pulp	Carboxylic acids	Lactic acid	26.88 ± 0.69 g L^−1^	50 mL phosphate buffer (50 mmol L^−1^, pH 6.5);10 mL MRS media; 6 g exhausted sugar beet pulp pellets (ESBPP) hydrolysates; 30 g L^−1^ CaCO_3_	*Lactobacillus casei* 2246	[79]
			50 g L^−1^	60 mL ESBP hydrolysates; [g L^−1^] 5 yeast extract; 18 CaCO_3_; pH 6.5	*Lactiplantibacillus plantarum* CECT 748	[80]
			30 g L^−1^	50 mL ESBPP hydrolysate; 5 g L^−1^ yeast extract; different concentrations of CaCO_3_ (9.0 or 18.0 or 27.0 g L^−1^)	*Lactobacillus plantarum* CECT 748	[81]
Sugarcane bagasse (SCB)	Biopolymer	Lasiodiplodan ((1→6)-β-d-glucan)	22.0 g L^−1^	50 mL of medium (40 g L^−1^ sugarcane bagasse cellulosic hydrolysate (SCBCH); 10 g L^−1^ rice bran extract (RBE))	*Lasiodiplodia theobromae* CCT 3966	[82]
			16.2 g L^−1^	50 mL of medium (40 g L^−1^ sugarcane bagasse cellulosic hydrolysate (SCBCH); 10 g L^−1^ soybean bran extract (SBE))		
Birch (*Betula pendula*)	Cell biomass	Yeast biomass	1.1 OD	Lignocellulosic hydrolysate-enriched C5-sugars. Dilutions made using minimal medium ([g L^−1^] monopotassium phosphate 3; magnesium sulfate, 0.5); C/N ratio adjusted by modifying concentration of added ammonium sulfate; pH 6.0	*Candida parapsilosis* DSM 70125	[10]
			1.0 OD		*Kluyveromyces marxianus* CBS 6556	
Sugarcane straw	Enzymes	Xylanase	90.2 U mL^−1^	[*m*/*v*]: 3.0% pretreated sugarcane straw; 0.1% (NH_4_)_2_SO_4_; 0.0017% MgSO_4_·7H_2_O; 0.1% K_2_HPO_4_; 0.0028% ZnSO_4_; 0.1% NH_4_H_2_PO_4_; 0.06% KCl; 0.1% yeast extract; 0.1% sucrose; pH 4.5	*Trichoderma reesei* QM9414	[72]
		Cellulase	0.5 FPU mL^−1^			
	Biopolymer	β-Glucan	4.7%	[g L^−1^]: SCS hydrolysate (40 glucose concentration); 2 KH_2_PO_4_; 2 MgSO_4_ · 7 H_2_O; 1 yeast extract; pH 7.0	*Lasiodiplodia theobromae* CCT3966	[57]
Wheat straw	Enzymes	Xylanase	53.7 U mL^−1^	[g L^−1^]: (NH_4_)_2_SO_4_ 1.3; KH_2_PO_4_ 0.37; MgSO_4_⋅7H_2_O 0.25; CaCl_2_⋅2H_2_O 0.07; FeCl_3_ 0.02; yeast extract 1.0; beechwood xylan 0.5% [*w*/*v*], 2% [*w*/*v*] agar	*Penicillium chrysogenum* A3 DSM105774	[83]
		Phytase	16.46 ± 0.56 U g^−1^ of dry substrate	Wheat straw 5 g dry weight; [g g^−1^ of dry substrate]: glucose 0.17; (NH_4_)_2_SO_4_ 0.068; [g kg^−1^] 655 moisture	*Aspergillus ficuum* PTCC 5288	[84]
Rice straw	Enzymes	Xylanase	6721.9 U g^−1^ of dry substrate	Rice straw waste 5 g; Mandel Weber medium (77.5% initial moisture content; [g L^−1^]: 1.4 (NH_4_)_2_SO_4_; 2.0 KH_2_PO_4_; 0.3 CaCl_2_; 0.3 MgSO_4_⋅7H_2_O; 0.02 Tween-80; 0.005 FeSO_4_⋅7H_2_O; 0.0016 MnSO_4_⋅7H_2_O; 0.0014 ZnSO_4_⋅7H_2_O; 0.002 CoCl_2_⋅6H_2_O)	*Schizophyllum commune* ARC-11	[73]
		Xylanase	2518.51 U mL^−1^	[g L^−1^]: 5 yeast extract; 1 peptone; 1 NaNO_3_; 1 KH_2_PO_4_; 0.02 MgSO_4_⋅7H_2_O; 10 rice straw	*Bacillus altitudinis* RS3025	[85]
		Cellulase	124.94 U g^−1^	3.75 g (1.5% *w*/*v*) of rice straw moistened with 11.25 mL (1:3 biomass to moistening agent) of modified Mandel’s medium ([g L^−1^]: (NH_4_)_2_SO_4_ 1.4; KH_2_PO_4_ 2; urea 0.63; CaCl_2_ 0.3; MgSO_4_⋅7H_2_O 0.3; peptone 0.75; 1 mL of trace element solution); pH 7	*Aspergillus terreus* RS2	[86]
	Polyhydroxy alcohols	Xylitol	25.8 g L^−1^	Rice straw hydrolysate [g L^−1^]: xylose 45.0; yeast extract 0.5; peptone 0.5; antifoam agent 60–100 μL; pH 5	*Candida tropicalis* MTCC 6192	[87]
Quinoa straw	Polyhydroxy alcohols	Xylitol	26.05 ± 0.31 (g L^−1^) (0.5 g g^−1^)	Detoxified hydrolysate; yeast extract 5 g L^−1^; tryptone 4 g L^−1^; pH 5.5	*Candida tropicalis* CICC 1779	[25]
Rapeseed straw	Polyhydroxy alcohols	Xylitol	0.55 g g^−1^	Detoxified hydrolysate [g L^−1^]: glucose 11.68 ± 0.09; xylose 40.59 ± 0.21; galactose 8.67 ± 0.12; arabinose 6.79 ± 0.09; mannose 2.22 ± 0.06	*Candida guilliermondii* FTI 20037 (ATCC 201 935)	[60]
			0.45 g g^−1^		*Debaryomyces hansenii* (NRRL Y-7426)	
Oat bran	Cell biomass	Yeast biomass	9.35 ± 0.55 g L^−1^	50 mL of hydrolysate; 6.7 g L^−1^ Yeast Nitrogen Base (Sigma-Aldrich).	*Yarrowia lipolytica* A101	[88]
Rice bran	Enzymes	Laccase	4.58 U mL^−1^	2 g rice bran; 100 mL mineral basal salt solution (MBSS) [g L^−1^]: dextrose 10.0; peptone 3.0; K_2_HPO_4_ 0.4; ZnSO_4_ 0.01; MnSO_4_ 0.5; KH_2_PO_4_ 0.6; FeSO_4_ 0.0005; MnSO_4_ 0.5	*Bacillus aquimaris* AKRC02	[89]
Corn cob	Enzymes	Xylanase	50.55 U mL^−1^	[g L^−1^]: corn cob 20.0; MgSO_4_ 2.0; NaNO_3_ 1.4; KH_2_PO_4_ 1.8; NH_4_Cl 2.0; CaCO_3_ 1.2	*Aspergillus fumigatus* SD5A	[90]
			48.63 U g^−1^		*Aspergillus fumigatus* L1	
Corn stover	Terpenes	Limonene	20.57 mg L^−1^	YPBiomass medium [g L^−1^]: 10 yeast extract; 20 peptone; 50% detoxified lignocellulosic hydrolysate (*v*/*v*)	Engineered *Yarrowia lipolytica*	[91]
		β-Farnesene	7.38 ± 0.24 g L^−1^	500 mL of the initial 100% lignocellulosic hydrolysate media (40.3 ± 0.4 g L^−1^ glucose, 14.7 ± 0.3 g L^−1^ xylose, 5 mM magnesium sulfate, pH 6.0)	Engineered *Yarrowia lipolytica* ATCC MYA2613	[92]
		1,8-Cineole	1.4 g L^−1^	Lignocellulosic hydrolysate derived from corn stover; 5 g L^−1^ ammonium sulfate; 100 µM iron sulfate; 100 mM potassium phosphate	*Rhodosporidium toruloides*	[93]
		α-Bisabolene	2.6 g L^−1^			
Corn stalk	Biopolymer	Bacterial cellulose	2.86 g L^−1^	Detoxified hydrolysate; [%] 0.5 baco-peptone; 0.5 yeast extract; 1.5 D-mannitol; 0.2 magnesium sulfate; 0.5 anhydrous ethanol; pH 68	*Acetobacter xylinum* ATCC 23767	[94]
Pineapple cores	Polyhydroxy alcohols	Xylitol	0.371 g g^−1^ glucose	100 mL of hydrolysate; 10 mL of tenfold-concentrated nutritional medium containing [g L^−1^]: 9.44 (NH_4_)_2_SO_4_; 2.5 KH_2_PO_4_; 0.5 MgSO_4_⋅7H_2_O; 0.05 CaCl_2_⋅2 H2O; 0.5 citric acid; 0.1 Myo-inositol; 0.035 FeSO_4_·7 H_2_O; 0.02 calcium pantothenate; 0.011 ZnSO_4_·7H_2_O; 0.0092 MnSO_4_·7H_2_O; 0.005 pyridoxal hydrochloride; 0.005 nicotine acid; 0.005 thiamine hydrochloride; 0.0035 KI; 0.002 CoCl_2_⋅6H_2_O; 0.002 H_3_BO_3_; 0.0013 Na_2_CoO_4_⋅2H_2_O; 0.001 CuSO_4_⋅7H_2_O; 0.001 aminobenzoic acid; 0.0005 Al_2_(SO_4_)_3_; 0.0001 D-biotin	*Candida tropicalis*	[95]
Potato pulp waste	Cell biomass	Fodder yeast biomass	39.3%	Potato pulp hydrolysate (200 g potato pulp; [mL]: 800 water; 0.05 Thermamyl; 0.1 San Extra; 0.1 Cellic CTec 2 (15 FPU)); (g L^−1^): 0.2 (NH_4_)_2_HPO_4_, 0.06 MgSO_4_⋅7H_2_O, pH 4.8–5.2	*Candida guilliermondii* ATCC 6260	[96]
Potato peel	Enzymes	α-Amylase	3014.30 U g^−1^	20 g raw potato peel; 2 mL salt solution [g L^−1^]: MgSO_4_ 2; KH_2_PO_4_ 10; MnSO_4_ 0.5; NaCl 2	*Aspergillus niger*	[97]
			1112.25 U g^−1^ of dry substrate	1 g potato peel; 1 mL liquid medium [%, *w*/*v*]: NaNO_3_ 0.3; MgSO_4_ 0.05; KCl 0.05; FeSO_4_ 0.002; K_2_HPO_4_ 0.1; pH 3.0	*Aspergillus niger* RBP7	[98]
			48.14 ± 0.43 U mL^−1^	5 g raw potato peel powder amended with 10 mL minimal salt solution [g L^−1^]: KH_2_PO_4_ 2.0; MgSO_4_⋅7H_2_O 0.2; NaCl 0.1; CaCl_2_ 0.1; MnSO_4_ 0.5; peptone 0.2; pH 6.0	*Aspergillus flavus* S2-OY	[99]
		Amylase	16.9 U mL^−1^	[g L^−1^]: 20 potato peel powder; 2 yeast extract; 5 peptones; 0.5 MgSO_4_; 0.5 KH_2_PO_4_; 1.5 NaCl; 0.5 CaCl_2_	*Bacillus aerius* FPWSHA	[100]
			64.9 U mL^−1^	[g L^−1^]: 10 potato peel powder; 1.5 KH_2_PO_4_; 0.5 MgSO_4_; 0.01 CaCl_2_; 0.003 FeSO_4_; pH 8.0	*Anoxybacillus rupiensis* T2	[101]
		Protease	12.3 U mL^−1^	[g L^−1^]: 20 potato peel powder; 2 yeast extract; 5 peptones; 0.5 MgSO_4_; 0.5 KH_2_PO_4_; 1.5 NaCl; 0.5 CaCl_2_	*Bacillus aerius* FPWSHA	[100]
			26.2 U mL^−1^	[g L^−1^]: 10 potato peel powder; 1.5 KH_2_PO_4_; 0.5 MgSO_4_; 0.01 CaCl_2_; 0.003 FeSO_4_; pH 8.0	*Anoxybacillus rupiensis* T2	[101]
		Pytase	138.4 U mL^−1^	[g L^−1^]: glucose 10.0; (NH_4_)_2_SO_4_ 3.0; KCl 0.5; MgSO_4_⋅7H_2_O 0.5; CaCl_2_ 0.1; calcium phytate 0.5%; pH 5.5	*Talaromyces purpureogenus* NSA20	[102]
	Biopolymer	Bacterial cellulose	4.7 g L^−1^	PPW acid hydrolysate; pH 6.0	*Gluconacetobacter xylinus*	[103]
Potato waste	Enzymes	Phytase	12.93 ± 0.47 U g^−1^	Potato waste (moisture content 79%); 4% (*w*/*w*) (NH_4_)_2_SO_4_	*Aspergillus ficuum* ATCC 6687	[104]
Sweet potato peel	Carboxylic acids	Citric acid	4.36 ± 006 mg mL^−1^	90 mL sweet potato peel starch hydrolysate; 10 mL nutrient solution [g L^−1^]: 2.23 NH_4_NO_3_; 0.23 MgSO_4_⋅7H_2_O; 1.0 KH_2_PO_4_; pH 6.5	*Aspergillus niger*	[105]
Papaya peels	Cell biomass/single-cell protein	Biomass	11.73 ± 0.81 g L^−1^	1000 mL fruit peel medium (10%, *v*/*v*): 100 mL fruit juice; inorganic supplements [g]: 1.0 KH_2_PO_4_; 0.5 MgSO_4_⋅7H_2_O; 0.1 NaCl; 0.1 CaCl_2_; 900 mL distilled water	Palmyrah toddy sample as the source of natural mixed culture of yeast and bacteria	[106]
		Single-cell protein (SCP)	52.4 ± 0.4%			
Pomegranate peel	Enzymes	Xylanase	1469.40 U mL^−1^	[g L^−1^]: NaNO_3_ 2.0; K_2_HPO_4_ 0.5; KCl 0.5; MgSO_4_ 0.7; H_2_O 0.5; pomegranate peel 20.0	*Chaetomium globosum*	[107]
	Carboxylic acids	Citric acid	306.8 g kg^−1^	10 g crushed pomegranate peels (0.5–1.0 cm diameter) without dryin;, 75% moisture content; pH 8.0; 3% (*w*/*w*) methanol	*Aspergillus niger* B60	[108]
	Polyhydroxy alcohols	Xylitol	55.57 g L^−1^	[g L^−1^]: detoxified hydrolysate about the content pomegranate peel 20; yeast extract 1.0; peptone 2.0; KH_2_PO_4_ 2.0; MgSO_4_·7 H_2_O 0.3; pH 7	*Candida tropicalis* LY15 (KJ734199)	[109]
Lemon peel	Enzymes	Total cellulase	10.96 ± 0.51 U mL^−1^	[g L^−1^]: lemon peel powder 0.5% *w*/*v*; urea 0.3; KH_2_PO_4_ 2.0; (NH_4_)_2_SO_4_ 1.4; MgSO_4_⋅7H_2_O 0.3; CaCl_2_⋅6H_2_O 0.3; FeSO_4_⋅7H_2_O 0.005; MnSO_4_ 0.002; ZnSO_4_ 0.002; CoSO_4_⋅7H_2_O 0.002; 2 mL L^−1^ Tween 80	*Trichoderma afroharzianum* NAS107	[110]
		Exoglucanase	5.42 ± 0.12 U mL^−1^			
		Endoglucanase	6.02 ± 0.19 U mL^−1^			
		β-Glucosidase	3.97 ± 0.15 U mL^−1^			
		Pectinase	1.62 ± 0.11 U mL^−1^			
		Xylanase	4.11 ± 0.49 U mL^−1^			
Mandarin peel	Oligosaccharides	Oligosaccharide	4.49 ± 0.48 mg mL^−1^	Citrus peel waste powder liquid medium (substrate concentration: 1–11% (*w*/*v*); 50 mM buffer (acetate buffer from 3.0 to 5.0; phosphate buffer from 6.0 to 8.0); 0.0004% biotin; 0.5% methanol); final culture volume 25 mL	*Pichia pastoris* X-33	[111]
Orange peel			1.99 ± 0.13 mg mL^−1^			
Pineapple peel	Polyhydroxy alcohols	Xylitol	0.31 g g^−1^	[g L^−1^] hydrolysate supplemented with 10 yeast extract (control)/urea; 2.0 (NH_4_)_2_SO_4_; 0.1 CaCl_2_⋅2H_2_O	*Candida tropicalis* FTI 20037	[112]
Kiwi peel	Biopolymer	Bacterial cellulose	11.53%	Peel hydrolysates 500 mL; 500 mL HS (Hestrin–Schramm) medium ([g L ^−1^]: 20 glucose; 5 yeast extract; 5 peptone; 2.7 Na_2_PO_4_; 1.15 citric acid)	*Komagataeibacter hansenii* GA2016	[113]

## Data Availability

No new data were created or analyzed in this study. Data sharing is not applicable to this article.
